# Supplementary material for: Splice-Junction-Based Mapping of Alternative Isoforms in the Human Proteome
Source: Cell Rep. Author manuscript; Available in PMC 2020 Jan 15. (PMC6961840; doi:10.1016/j.celrep.2019.11.026)
Supplement: 2 [file NIHMS1546469-supplement-2.zip › DF1/PXD010154/Heart/Heart_1_TTN_KPEAPPPKVPEVLPPKEVVPEK.pdf]

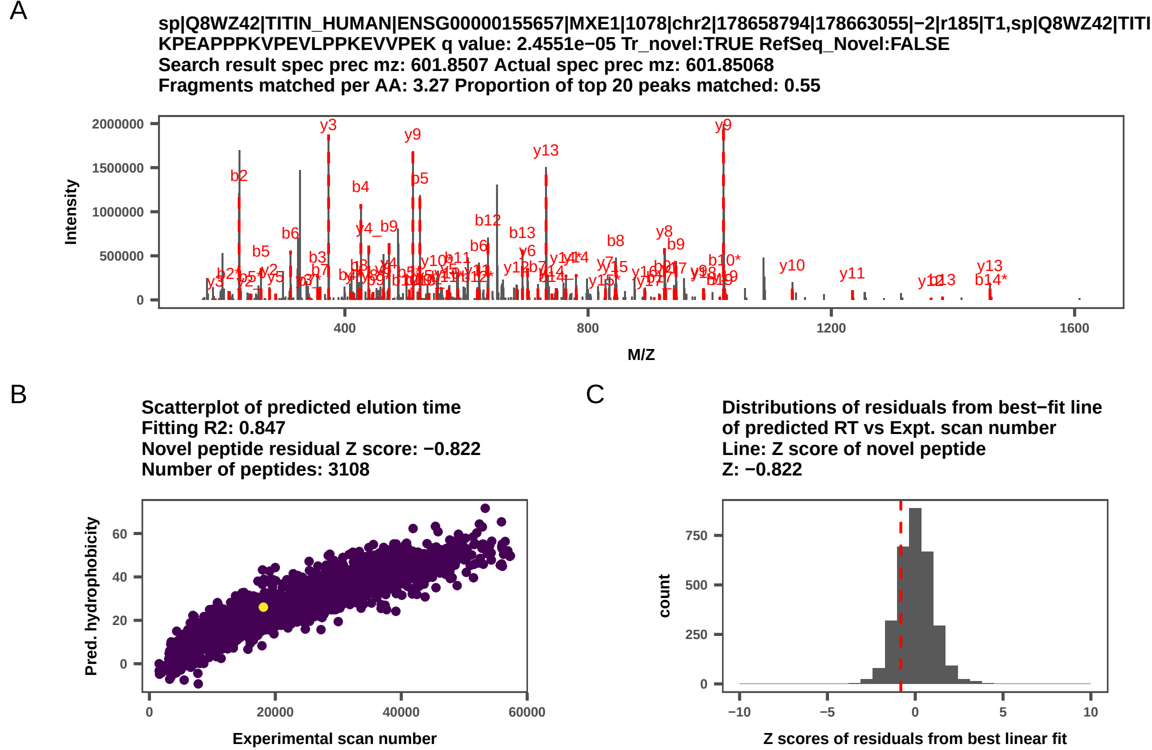

A
sp|Q8WZ42|TITIN_HUMAN|ENSG00000155657|MXE1|1078|chr2|178658794|178663055|−2|r185|T1,sp|Q8WZ42|TITI
KPEAPPPKVPEVLPPKEVVPEK q value: 2.4551e−05 Tr_novel:TRUE RefSeq_Novel:FALSE
Search result spec prec mz: 601.8507 Actual spec prec mz: 601.85068
Fragments matched per AA: 3.27 Proportion of top 20 peaks matched: 0.55
Intensity
2000000
1500000
1000000
500000
0
400
800
1200
1600
M/Z
B
Scatterplot of predicted elution time
Fitting R2: 0.847
Novel peptide residual Z score: −0.822
Number of peptides: 3108
Pred. hydrophobicity
60
40
20
0
0
20000
40000
60000
Experimental scan number
C
Distributions of residuals from best−fit line
of predicted RT vs Expt. scan number
Line: Z score of novel peptide
Z: −0.822
count
750
500
250
0
−10
−5
0
5
10
Z scores of residuals from best linear fit
